# The effect of binaural beat audio on anxiety in patients undergoing fiberoptic bronchoscopy: A prospective randomized controlled trial

**DOI:** 10.1097/MD.0000000000029392

**Published:** 2022-06-17

**Authors:** Pornchai Opartpunyasarn, Pornpattana Vichitvejpaisal, Nittha Oer-areemitr

**Affiliations:** a Pulmonary and Critical Care Division, Department of Medicine, Phramongkutklao Hospital, Bangkok, Thailand; b Department of Medicine, Chulabhorn Hospital, Bangkok, Thailand; c Department of Ophthalmology, Chulabhorn Hospital, Bangkok, Thailand.

**Keywords:** anxiety, binaural beat, bronchoscopy, music

## Abstract

**Background:**

Fiberoptic bronchoscopy is an invasive procedure known to induce anxiety in patients. Binaural beat therapy, in which sounds of different frequencies are delivered to the 2 ears to entrain the brainwaves, has been used to reduce anxiety in some operations. This study aimed to determine the anxiolytic effects of binaural beat audio in patients undergoing fiberoptic bronchoscopy.

**Methods:**

Eligible subjects were randomly assigned to receive binaural beat music, plain music, or no music. They were asked to wear earphones starting approximately 15 minutes before the bronchoscopy. The level of anxiety was measured using the State-Trait Anxiety Inventory questionnaire. Blood pressure, heart rate, and sedative drug administration were also recorded.

**Results:**

One hundred and twelve subjects were randomized into binaural beat music (n = 38), plain music (n = 38), and no music (n = 36) groups. The mean change in post-bronchoscopy State-Trait Anxiety Inventory state score in the binaural beat music, plain music, and no music group was −7.26 (*P* < .001), −3.92 (*P* = .005), and −1.12 (*P* = .454), respectively. The mean systolic blood pressure and diastolic blood pressure significantly decreased from baseline by −9.89 (*P* = .002) and −5.76 (*P* = .005), respectively, in the binaural group. The mean heart rate increased from baseline by 3.32 (*P* = .035), 5.21 (*P* = .038), and 3.64 (*P* = .149) in the binaural beat music, plain music, and no music groups, respectively.

**Conclusion:**

Binaural beat music appeared to reduce anxiety among patients undergoing fiberoptic bronchoscopy.

Trial registration: TCTR, TCTR20200915002. Registered 14 September 2020 - Retrospectively registered.

## 1 Introduction

Fiberoptic bronchoscopy is a diagnostic and therapeutic respiratory procedure in which the inner airways are visualized using a camera. The procedure may include the collection of samples of lung tissue, bronchoalveolar lavage, and the removal of foreign bodies or mucus plugs. A bronchoscopist is tasked with inserting the bronchoscope tube into the airways, usually through the nose or mouth, or occasionally through a tracheostomy. Most patients report feeling worried that the procedure will be painful, and may exhibit aspiration, breathing difficulties, and complications. Accordingly, conscious sedation with opioids and/or benzodiazepines has been used to relieve patient discomfort and anxiety during the procedure.^[2–4]^ However, high doses of these drugs can lead to unfavorable side effects, such as cardiovascular and respiratory suppression.

Music therapy has been suggested as a safe and affordable non-pharmacological means of reducing stress and anxiety in patients through a number of different interventional procedures.^[5,6]^ Previous studies have demonstrated beneficial outcomes of music therapy, including a shorter operative time, decreased sedative drug use, and lower levels of pain.^[6–8]^ Wilson's meta-analysis revealed that listening to music during a bronchoscopy reduced blood pressure and heart rate among patients.^[9]^ Listening to self-selected music prior to bronchoscopy reduces anxiety in patients with suspected lung cancer reported by Jeppesen et al.^[10]^ Musical sounds may affect emotional and physiological brain processes related to perioperative anxiety (e.g., blood pressure and heart rate). In particular, music is known to control the release of cortisol, which is an essential hormone implicated in stress responses.^[11]^

Over 40 years ago, Gerald Oster described binaural beat music as a special type of audio.^[12]^ Neuropsychologists and clinicians have increasingly considered binaural beat music to be a therapeutic tool with potential in decreasing perioperative anxiety.^[13,14]^ Binaural beat music comprises 2 audio waves with different frequencies that are separately presented to each ear. The frequencies of these waves must be lower than 1000 hertz (Hz), and it is recommended that the frequencies of the 2 pitches be within 30 Hz. For instance, for tones with frequencies of 450 Hz and 460 Hz delivered to the left and the right ears via headphones, the binaural beat frequency would be 10 Hz. This is in the alpha range of brainwaves, which is associated with relaxation.

In the present study, we compared the anxiolytic effects of binaural beat audio, music without a binaural beat, and no musical intervention on patients undergoing fiberoptic bronchoscopy.

## 2 Materials and methods

### 2.1 Study design

This was a prospective, randomized, double-blind, controlled trial. The study protocol was approved by the Institutional Review Board of the Royal Thai Army Medical Department at Phramongkutklao College of Medicine. Written informed consent was obtained from all subjects after the receipt of written information about the study, and all participants had the opportunity to ask the investigators questions regarding the study protocol.

### 2.2 Subjects

Patients aged ≥18 years were recruited and scheduled for elective fiberoptic bronchoscopy under intravenous sedation at the Pulmonary and Critical Care Division of Phramongkutklao Hospital, Thailand, from May 2018 to July 2019. Those with hearing impairments, ear infections, a history of epilepsy, cognitive dysfunction, cardiac arrhythmia, and blood pressure <90/60 mm Hg or >160/100 mm Hg were excluded from participation.

### 2.3 Randomization

Eligible subjects were randomized into the following 3 groups using a block randomization technique (blocks of 6 participants): a binaural group (music with a binaural beat), plain music group (identical to the first group but without a binaural beat), and a control group (earphones with no music). The bronchoscopists and investigators who played the mp3 audio file during the study were blinded to the audio type. The subjects were also blinded to their group assignment.

### 2.4 Interventions

The binaural beat audio stimulus was synthesized using the Self Hypnosis and Relaxation Machine (S.H.A.R.M., CyberTeam, Ltd., Informer Technologies Inc., Madrid, Spain), version 2.4, with carrier tones at 109 and 209 Hz. The stimulus had a frequency of 20 Hz during the first 5 minutes, and then gradually declined to the therapeutic frequency 10 Hz within the next 5 minutes. This was then continually maintained for up to 50 minutes. The 60-minute binaural beat composition included relaxing melodies, tones, and rhythms with added nature sounds such as the sound of a waterfall, birds chirping, the ocean, rivers, and a forest. The stimulus was generated as a high quality MP3 format. In the plain music group, the stimulus was the same, except the binaural beat was absent. We verified that the binaural beat was difficult to detect in the experimental stimulus.

All bronchoscopic procedures were performed with the participant in the supine posture. Before the procedure, each subject was assigned to receive 1 audio file according to the randomization process. An MP3 player and earphones (Remax Sport Wired Headset: RM-S15) were given to each participant, including those in the silent control group. The earphones were placed in both ears about 15 minutes before the bronchoscopy and were not removed until 15 minutes after the complete procedure. The sound volume was set at a level that did not interfere with communication between the patient and bronchoscopist. The bronchoscopist was not aware of the group assignment of the participant.

### 2.5 Assessment of anxiety

The effect of the binaural beat audio on anxiety status was examined using the State-Trait Anxiety Inventory (STAI) questionnaire. Anxiety was measured using the state subscale (STAI-S) and the trait subscale (STAI-T). The STAI-S comprises 20 questions designed to test how subjects “feel right now,” and the STAI-T has 20 questions that assess how the subjects “generally feel.” The scores for both subscales range from 20 to 80 with higher scores corresponding to higher levels of anxiety.^[15–17]^ The subjects were asked to complete both the STAI-S and STAI-T questionnaires on the day of the bronchoscopy before inserting the earphones. The STAI-S was then completed for a second time after the bronchoscopic procedure.

We used blood pressure and heart rate as objective measures of anxiety. Systolic blood pressure (SBP), diastolic blood pressure (DBP), and heart rate (HR) were measured before earphone insertion and 15 minutes after the bronchoscopy. The amounts of sedatives and analgesics used were recorded.

### 2.6 Outcomes

The primary outcome was changes in anxiety level as measured by the STAI-S before and after the bronchoscopy. The secondary outcomes were changes in the SBP, DBP, and HR after the bronchoscopy and the total amounts of sedatives and analgesic drugs used during the procedure. The duration of the procedure and intra-bronchoscopic adverse events were also recorded.

### 2.7 Statistical analysis

According to the data from previous studies,^[14]^ a sample size of 27 participants in each group was required to provide a power level of 90% and type I error probability of 5%. Statistical analysis was performed using SPSS version 23.0, and we used a one-way ANOVA for continuous variables and a Chi-Squared test for categorical variables. Paired *t*-tests and Bonferroni tests were used to examine the differences within and between the groups, respectively. A *P* value ≤.05 was considered statistically significant.

## 3 Results

**Figure 1:**
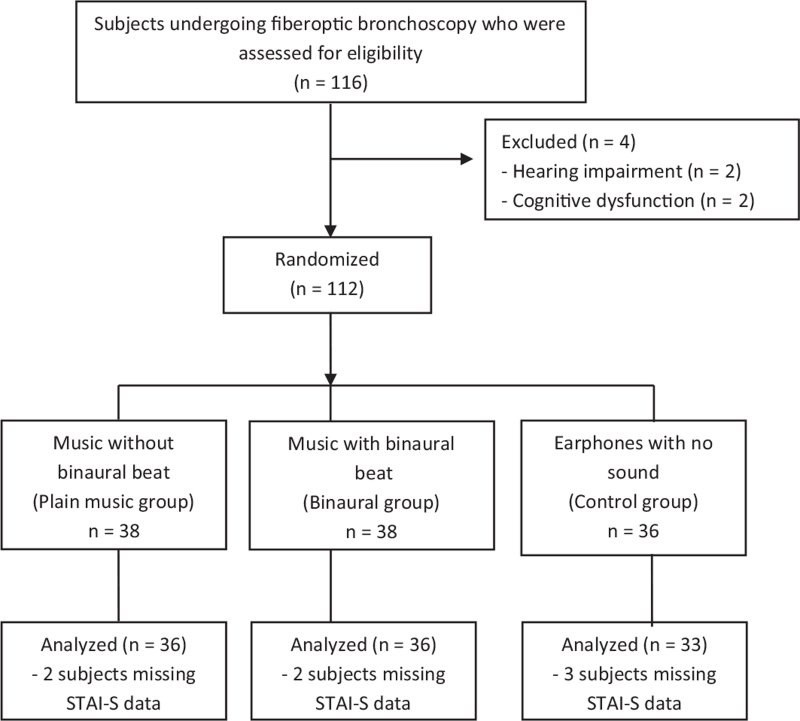
CONSORT flow diagram of study subjects. STAI-S = State-Trait Anxiety Inventory-State.

From May 2018 to July 2019, 116 subjects were scheduled for the bronchoscopic procedure and included in the study. Of these, 4 subjects were excluded from analysis because of hearing impairments (n = 2) and cognitive dysfunction (n = 2). The remaining 112 subjects were randomized into the binaural beat music (n = 38), the plain music (n = 38), and the control (n = 36) groups. Overall, 7 subjects (2 in the plain music group, 2 in the binaural group, and 3 in the control group) did not complete the STAI-S questionnaire (Fig. 1). There were no significant differences in demographic data and covariant factors between the groups (Table 1).

**Table 1:** Demographic data.

Variable	Plain music (n = 38)	Binaural (n = 38)	Control (n = 36)	*P* value
Age	58.05 ± 11.99	57.76 ± 13.34	56.72 ± 14.13	.902
Sex				
Male	27 (71.1%)	21 (55.3%)	21 (58.3%)	.326
Underlying disease				
DM	3 (7.9%)	6 (15.8%)	10 (27.8%)	.073
HT	12 (31.6%)	13 (34.2%)	12 (33.3%)	.970
Cardiovascular disease	1 (2.6%)	1 (2.6%)	3 (8.3%)	.394
Respiratory disease	6 (15.8%)	8 (21.1%)	1 (2.8%)	.061
Malignancy	10 (26.3%)	10 (26.3%)	9 (25%)	.989
Other	12 (31.6%)	12 (31.6%)	7 (19.4%)	.407
Drug use				
Beta blocker	1 (2.6%)	2 (5.3%)	2 (5.6%)	.796
Sedative or anxiolytic	6 (15.8%)	6 (15.8%)	4 (11.1%)	.804
Previous bronchoscopy				
0	24 (63.2%)	26 (68.4%)	21 (58.3%)	.686
1	11 (28.9%)	9 (23.7%)	9 (25%)	
≥2	3 (7.9%)	3 (7.9%)	6 (16.7%)	
Procedure				
EBUS-GS	21 (55.3%)	21 (55.3%)	14 (38.9%)	.450
TBB	7 (18.4%)	5 (13.2%)	7 (19.4%)	
EBUS-TBNA	7 (18.4%)	7 (18.4%)	8 (22.2%)	
EBUS-GS &EBUS-TBNA	2 (5.3%)	1 (2.6%)	5 (13.9%)	
TBNA	0 (0%)	2 (5.3%)	0 (0%)	
Airway surveillance	1 (2.6%)	2 (5.3%)	2 (5.6%)	
STAI-T scores	34.55 ± 8.34	36.45 ± 8.77	34.81 ± 7.84	.564
Operative time (min)	48.42 ± 22.39	50.26 ± 21.93	58.19 ± 26.57	.176
Final diagnosis				
Non-small cell lung cancer	8 (21.1%)	14 (36.8%)	13 (36.1%)	.643
Tuberculosis	6 (15.8%)	5 (13.2%)	4 (11.1%)	
Undiagnosed	20 (52.6%)	13 (34.2%)	15 (41.7%)	
Other	4 (10.5%)	6 (15.8%)	4 (11.1%)	

Values are presented as mean ± SD and number (%). DM = diabetes mellitus, EBUS-GS = endobronchial ultrasonography with guide sheath, EBUS-TBNA = endobronchial ultrasound-guided transbronchial needle aspiration, HT = hypertension, STAI-T = State-Trait Anxiety Inventory-Trait, TBB = transbronchial biopsy, TBNA = transbronchial needle aspiration.

The bronchoscopic procedures were performed by 13 different physicians assisted by 7 different nurses. No statistically significant differences were observed between the groups regarding the number of attending staff members, operative time, or the procedures performed during the bronchoscopy (Table 1). No major adverse events were reported.

There were no significant differences in STAI-T scores between the groups (Table 1). The baseline STAI-S scores were 39.26 ± 8.94, 35.62 ± 8.45, and 35.64 ± 10.57 for the binaural beat music, the plain music, and the control groups, respectively (*P* = .158). Differences in baseline SBP, DBP, and HR were also non-significant between the groups (SBP: 133.95 ± 22.49, 134.03 ± 19.31, and 133.03 ± 19.07, *P* = .973; DBP: 78.92 ± 11.88, 78.95 ± 9.01, and 76.33 ± 7.63, *P* = .419; HR: 80.29 ± 13.44, 84.34 ± 14.59, and 82.94 ± 16.8, *P* = .490 in the binaural beat music, the plain music, and the control groups, respectively) (Table 2).

**Table 2:** STAI-S score, SBP, DBP, HR, sedatives, and analgesics used.

Variable	Plain music (n = 38)	Binaural (n = 38)	Control (n = 36)	*P* value
STAI-S scores				
Pre	35.62 ± 8.45	39.26 ± 8.94	35.64 ± 10.57	.158
Post	32.03 ± 8.13	33.09 ± 7.34	34.79 ± 8.82	.360
Mean change (95% CI)	−3.92 (−6.58, −1.25)	−7.26 (−10.22, -4.3)	−1.12 (−4.13, 1.89)	.012
*P* value (within group)	.005	<.001	.454	
SBP				
Pre	134.03 ± 19.31	133.95 ± 22.49	133.03 ± 19.07	.973
Post	138.71 ± 19.49	124.05 ± 19.13	130.25 ± 20.84	.007
Mean change (95% CI)	4.68 (−2.41, 11.78)	−9.89 (-15.76, −4.03)	−2.78 (−9.35, 3.8)	.007
*P* value (within group)	.189	.002	.397	
DBP				
Pre	78.95 ± 9.01	78.92 ± 11.88	76.33 ± 7.63	.419
Post	78.42 ± 9.21	73.16 ± 10.4	78.36 ± 11.26	.043
Mean change (95% CI)	−0.53 (−4.15, 3.09)	−5.76 (−9.64, −1.89)	2.03 (−2.2, 6.25)	.017
*P* value (within group)	0.77	0.005	0.336	
HR				
Pre	84.34 ± 14.59	80.29 ± 13.44	82.94 ± 16.8	.490
Post	89.55 ± 15.77	83.61 ± 15.34	86.58 ± 14.12	.234
Mean change (95% CI)	5.21 (0.31, 10.11)	3.32 (0.24, 6.39)	3.64 (-1.37, 8.65)	.802
*P* value (within group)	0.038	0.035	0.149	
Fentanyl (mcg)	86.49 ± 38.91	91.45 ± 43.99	96.53 ± 49.7	.628
Midazolam (mg)	3.43 ± 1.48	3.61 ± 1.52	3.75 ± 1.65	.681

Values are presented as mean ± SD and mean change (95% CI). DBP = diastolic blood pressure, HR = heart rate, SBP = systolic blood pressure, STAI-S = state-trait anxiety inventory-state.

**Figure 2:**
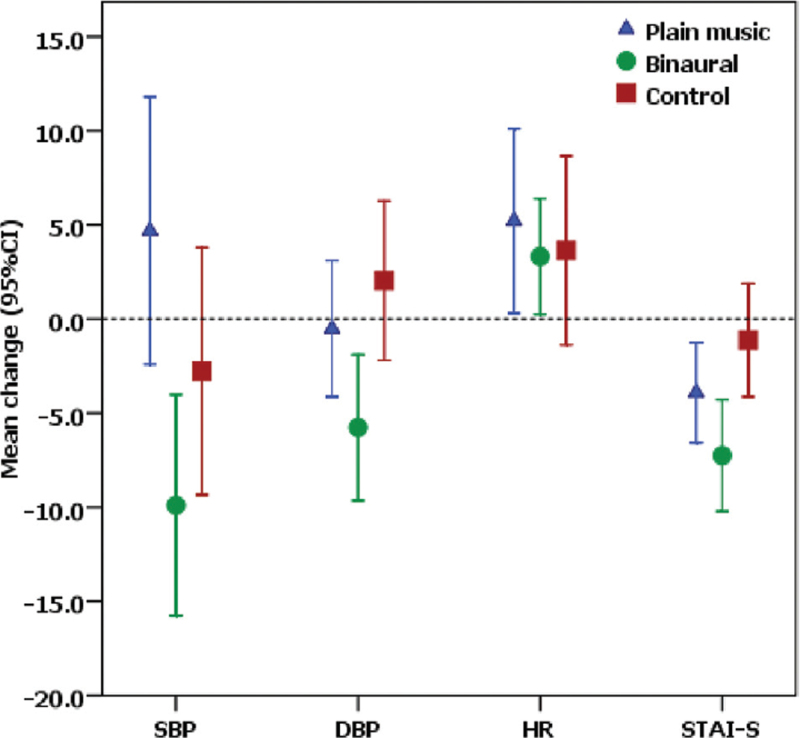
Mean change in SBP, DBP, HR, and STAI-S. DBP = diastolic blood pressure, HR =  heart rate, SBP = systolic blood pressure, STAI-S = state-trait anxiety inventory-state.

In terms of postbronchoscopic STAI-S scores, the mean change in STAI-S score was −7.26 (95% CI −10.22 to −4.3, *P* < .001) in the binaural beat music group, −3.92 (95% CI −6.58 to −1.25, *P* = .005) in the plain music group, and −1.12 (95% CI −4.13 to 1.89, *P* = .454) in the control group. The mean SBP in the binaural beat music group significantly decreased from baseline by −9.89 (95% CI −15.76 to −4.03, *P* = .002), as did the DBP by −5.76 (95%CI −9.64 to −1.89, *P* = .005), while no significant changes were found in the plain music and the control groups. The mean HR was increased from baseline by 3.32 (95% CI 0.24 to 6.39, *P* = .035) in the binaural beat music group, 5.21 (95% CI 0.31 to 10.11, *P* = .038) in the plain music group, and 3.64 (95% CI −1.37 to 8.65, *P* = .149) in the control group (Table 2 and Fig. 2). We compared the mean changes in STAI-S scores, SBP, DBP, and HR between the groups (Table 3). The binaural beat music group showed a significant reduction in STAI-S scores (*P* = .009) and DBP (*P* = .016) compared with the control group. Compared with the plain music group, the binaural beat music group showed a significant reduction in SBP (*P* = .005). The number of sedatives and analgesic drugs administered was non-significantly different between the groups (Table 2).

**Table 3:** Comparison of mean change in STAI-S, SBP, DBP, and HR between groups.

	Mean change (95% CI)			*P* value		
Variable	Plain music (n = 38)	Binaural (n = 38)	Control (n = 36)	Plain music vs Binaural	Plain music vs Control	Binaural vs Control
STAI-S	−3.92 (−6.58, −1.25)	−7.26 (−10.22, −4.3)	−1.12 (−4.13, 1.89)	.282	.5	.009
SBP	4.68 (−2.41, 11.78)	−9.89 (−15.76, −4.03)	−2.78 (−9.35, 3.8)	.005	.318	.369
DBP	−0.53 (−4.15, 3.09)	−5.76 (−9.64, −1.89)	2.03 (−2.2, 6.25)	.165	1	.016
HR	5.21 (0.31, 10.11)	3.32 (0.24, 6.39)	3.64 (−1.37, 8.65)	1	1	1

Values are presented as mean change (95% CI). DBP = diastolic blood pressure, HR = heart rate, SBP = systolic blood pressure, STAI-S = state-trait anxiety inventory-state.

## 4 Discussion

Our data indicate that binaural beat music could effectively reduce anxiety in individuals undergoing fiberoptic bronchoscopy, as we saw significant decreases in STAI-S scores, systolic blood pressure, and diastolic blood pressure. The findings are corresponded with our preliminary report data in ERS International Congress 2019.^[1]^ Prior studies have reported the benefits of binaural beat audio in other procedures. For instance, Padmanabhan et al showed that binaural beat audio delivered before general anesthesia decreased STAI-S scores in patients about to undergo surgery.^[13]^ Wiwatwongwana et al conducted a study in patients undergoing cataract surgery, and found that binaural beat music led to reduced STAI-S scores, systolic blood pressure, and heart rate.^[14]^ Despite studies demonstrating anxiolytic effects of music for bronchoscopy,^[9,10]^ none has been conducted using binaural beat music for this purpose. To the best of our knowledge, this is the first study to evaluate the effect of binaural beat audio on perioperative anxiety during bronchoscopy.

The STAI is currently a standard tool for measuring preoperative anxiety.^[15]^ In this study, we used the STAI as a subjective measure of anxiety, established via self-assessment. We also used objective parameters of anxiety, including SBP, DBP, HR, and sedative drug administration. We found a significant reduction in SBP, DBP, and STAI-S scores in the binaural beat music group compared with the plain music and control groups. However, we identified no significant effects of the treatment on HR. Nonetheless, our results suggest that binaural beat audio may have an additional anxiolytic effect beyond that of the plain musical intervention.

Binaural beat audio has been found to induce several predictable changes in brainwave activity. Specific binaural beat frequencies can induce different levels of brain activation according to the activity of the reticular-thalamic system. This process has been called “entrainment” in previous electroencephalography (EEG) studies.^[18]^ In the present study, we sought to assess the efficacy of binaural beat audio with a frequency of 10 Hz in reducing perioperative anxiety. Initially, a 20-Hz frequency was presented during the first 5 minutes in accordance with brainwaves corresponding to arousal. This was followed by a 10 Hz binaural beat to correspond with the enhancement of alpha waves associated with a relaxed mental state.

This study has some limitations. First, we did not objectively test for hearing impairment in the subjects, but rather relied on self-report. Thus, individuals with mild hearing loss who were not aware of their impairment could have participated in the study. In addition, subjects in the control group were only blinded until the intervention began. Awareness regarding group assignment could have led to some bias in terms of the post intervention STAI questionnaires. However, we anticipated that the physiological parameters of anxiety, including SBP, DBP, and HR, would reflect the anxiety state of the subjects. Another limitation is that we were unable to collect EEG recordings during the procedure to verify mental state. Further studies that include brainwave assessment via EEG monitoring are needed. Other potential clinical measures of the effects of binaural beat audio on perioperative anxiety, such as oxygen saturation, cough events, and procedure outcomes, could also be evaluated in future research.

## 5 Conclusions

Our data indicate that binaural beat audio can decrease the level of anxiety during a bronchoscopic procedure, as measured by STAI questionnaires. We found that physiological indicators of anxiety, such as systolic and diastolic blood pressure, were significantly lower in the binaural beat music group. Hence, music therapy with binaural beat audio has the potential to relieve anxiety in patients undergoing fiberoptic bronchoscopy.

## Acknowledgments

We are grateful to the participants for their involvement in this study. We also thank Sydney Koke, MFA, from Edanz Group (https://en-author-services.edanz.com/ac) for editing a draft of this manuscript.

## Author contributions

**Conceptualization:** Nittha Oer-areemitr, Pornchai Opartpunyasarn, Pornpattana Vichitvejpaisal.

**Data curation:** Pornchai Opartpunyasarn.

**Formal analysis:** Nittha Oer-areemitr, Pornchai Opartpunyasarn, Pornpattana Vichitvejpaisal.

**Investigation:** Nittha Oer-areemitr, Pornchai Opartpunyasarn, Pornpattana Vichitvejpaisal.

**Methodology:** Nittha Oer-areemitr, Pornchai Opartpunyasarn, Pornpattana Vichitvejpaisal.

**Resources:** Nittha Oer-areemitr, Pornchai Opartpunyasarn.

**Software:** Pornpattana Vichitvejpaisal.

**Writing – original draft:** Nittha Oer-areemitr, Pornchai Opartpunyasarn, Pornpattana Vichitvejpaisal.

**Writing – review & editing:** Nittha Oer-areemitr, Pornchai Opartpunyasarn, Pornpattana Vichitvejpaisal.
